# Fetal congenital gastrointestinal obstruction: prenatal diagnosis of chromosome microarray analysis and pregnancy outcomes

**DOI:** 10.1186/s12884-023-05828-7

**Published:** 2023-07-08

**Authors:** Mengyao Ni, Xiangyu Zhu, Wei Liu, Leilei Gu, Yujie Zhu, Peixuan Cao, Yan Gu, Yan Xu, Chenyan Dai, Xing Wu, Ying Yang, Chunxiang Zhou, Jie Li

**Affiliations:** Center for Obstetrics and Gynecology, Nanjing Drum Tower Hospital, The Affiliated Hospital of Medical School, Nanjing University, Nanjing, China

**Keywords:** Chromosomal microarray analysis, Congenital gastrointestinal obstruction, Pregnancy outcomes, Prenatal diagnosis

## Abstract

**Objective:**

The aim of this study was to investigate the incidence of chromosome anomalies in different types of congenital gastrointestinal obstruction and assess pregnancy outcomes of fetuses with congenital gastrointestinal obstruction.

**Methods:**

A total of 64 cases with gastrointestinal obstruction between January 2014 and December 2020 were enrolled in this study. They were divided into three groups according to sonographic images. Group A: isolated upper gastrointestinal obstruction; Group B: isolated lower gastrointestinal obstruction; Group C: non-isolated gastrointestinal obstruction. The rate of chromosome anomalies in different groups was calculated. Pregnant women with amniocentesis were followed up by medical records and telephone. The follow-up included pregnancy outcomes and development of the live born infants.

**Result:**

From January 2014 to December 2020, there were 64 fetus with congenital gastrointestinal obstruction underwent chromosome microarray analysis(CMA), the overall detection rate of CMA testing was 14.1%(9/64). The detection rate of Group A, B and C were 16.2%, 0 and 25.0% respectively. 9 fetuses with abnormal CMA results were all terminated. Among 55 fetuses with normal chromosomes, 10(18.2%) fetuses were not found to have any gastrointestinal obstruction after birth. 17(30.9%) fetuses were diagnosed with gastrointestinal obstruction and underwent surgical treatment after birth, one of which had lower gastrointestinal obstruction combined with biliary obstruction and died due to liver cirrhosis. 11(20.0%) pregnancy were terminated due to multiple abnormalities. 5(9.1%) fetuses were intrauterine death. 3(5.5%) fetuses were neonatal deaths. 9(16.4%) fetuses were lost to follow-up.

**Conclusion:**

It is crucial to understand whether the gastrointestinal tract abnormality is isolated or associated to other findings. The risk of chromosomal abnormalities in fetuses with isolated lower gastrointestinal obstruction is lower than upper gastrointestinal obstruction. While genetic abnormalities excluded, a promising prognosis is expected for fetuses with congenital gastrointestinal obstruction.

## Introduction

Congenital gastrointestinal obstruction is a common digestive tract malformation in neonates, with an incidence of 0.7–4.2 per 10,000 live births [1–3]. Congenital gastrointestinal obstruction may have some ultrasound features during pregnancy and the most common is the “double bubble” sign. As fetal gastrointestinal tract(GIT) is the main rout to absorb amniotic fluid, polyhydramnios were often observed in fetus with gastrointestinal obstruction [4, 5]. These ultrasound phenotype of fetal gastrointestinal obstruction is usually detected in second or third trimester [6]. Genetic abnormalities, embryonic teratogens and infection are suspected as high risk factors associated with congenital gastrointestinal obstruction.

Previous studies find that gastrointestinal obstruction was noticed in 3–5% trisomy 21 [7, 8]. Other microdeletions, such as 17q12 deletion and 4q22.3 deletion, have been reported in fetus with gastrointestinal obstruction [4, 9]. However, the data on the incidence of genetic findings in fetus with gastrointestinal obstruction was limited. Moreover, researches on the outcome of these babies after birth were rare.

To promote data based genetic counseling, we investigated the incidence of chromosome anomalies in different types of congenital gastrointestinal obstruction, followed by evaluating the pregnancy outcomes of fetuses with congenital gastrointestinal obstruction.

## Materials and methods

### Subjects

From January 2014 to December 2020, 64 fetuses with congenital gastrointestinal obstruction suggested by ultrasound were tested by chromosome microarray analysis(CMA). The sample types included amniocytes or products of conception (POC) if the pregnancy was terminated before genetic testing. Parental peripheral blood samples were obtained along with fetal samples. Our study was approved by the Ethic Committee of the Nanjing Drum Town Hospital (No.2019–084-01). All participants signed informed consent.

### Group

They were divided into three groups according to sonographic images. Group A: isolated upper gastrointestinal obstruction; Group B: isolated lower gastrointestinal obstruction; Group C: non-isolated gastrointestinal obstruction.

### DNA extraction and CMA

Genomic DNA was extracted from amniotic fluid cells with Biochain Amniotic Fluid Genomic DNA Kit (BioChain Institute, Hayward, CA), from POC samples with QIAamp® DNA Mini kit (Qiagen, Inc., Hilden, Germany), and from peripheral blood with QIAamp® DNA Blood Mini Kit (Qiagen, Inc., Hilden, Germany). Seven short tandem repeat loci with high polymorphism, including *D2S1338*、*D21S11*、*D7S820*、*D13S317*、*D16S539*、*D18S51* and *AMXY* gene, were selected to identify maternal blood contamination by linkage analysis [10]. CMA was performed using the ThermoFisher CytoScan platform. All DNA samples were digested, amplified, fragmented, labeled and hybridized to CytoScan 750 K chips according to the manufacturer’s protocol. Raw data were analyzed by ChAS 3.1 software (ThermoFisher, USA).

Interpretation of the copy number variations(CNVs) was defined according to the American College of Medical Genetics and Genomics (ACMG) guidelines [11]. In our study, variants of unknown clinical significance (VOUS) were further tested by quantitative fluorescent polymerase chain reaction(PCR). If it was constitutive in phenotypically normal parent, the CNV was classified as “normal” [10].

### Genetic counseling and follow-up

Detailed genetic counseling was offered to all of the couples. Sixty-two pregnant women with amniocentesis were followed up by medical records and telephone. The clinical follow-up included pregnancy outcomes and development of the surviving infants at least one year old.

## Results

**Fig. 1:**
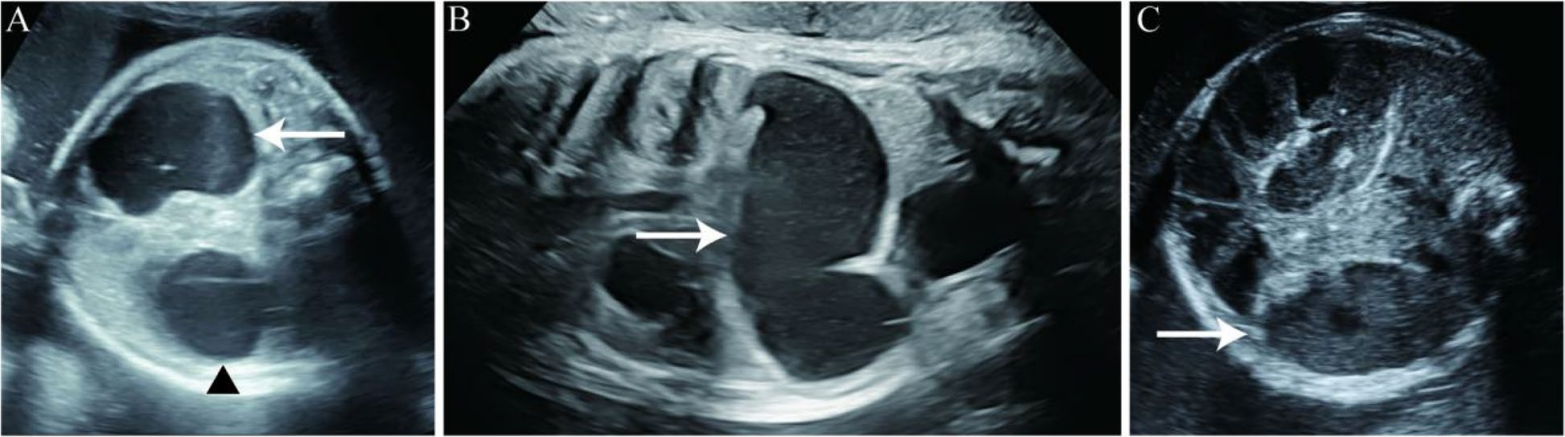
Representative prenatal ultrasound images from cases in the study. **A** Prenatal sonographic evaluation demonstrated a typical fetal double-bubble signal (black triangle: stomach, arrow: duodenum); **B** Coronal plane of intestinal dilatation (arrow: dilated bowel); **C** Cross section of intestinal dilation (arrow: dilated bowel)

Sixty-four singleton fetuses diagnosed with congenital gastrointestinal obstruction were enrolled between January 2014 and December 2020. The typical ultrasound images of congenital gastrointestinal obstruction were shown in Fig. 1. In our study, the gestational ages for diagnosing congenital gastrointestinal obstruction ranged from 23 to 36 weeks. The numbers of Group A, Group B and Group C were 37, 15 and 12 respectively.

### CMA results

Among the 64 cases, we found four cases of trisomy 21, three cases with pathogenic CNVs and two cases with both chromosome microdeletion and microduplication. The overall detection rate of CMA testing for fetuses with congenital gastrointestinal obstruction was 14.1% (9/64).

**Fig. 2:**
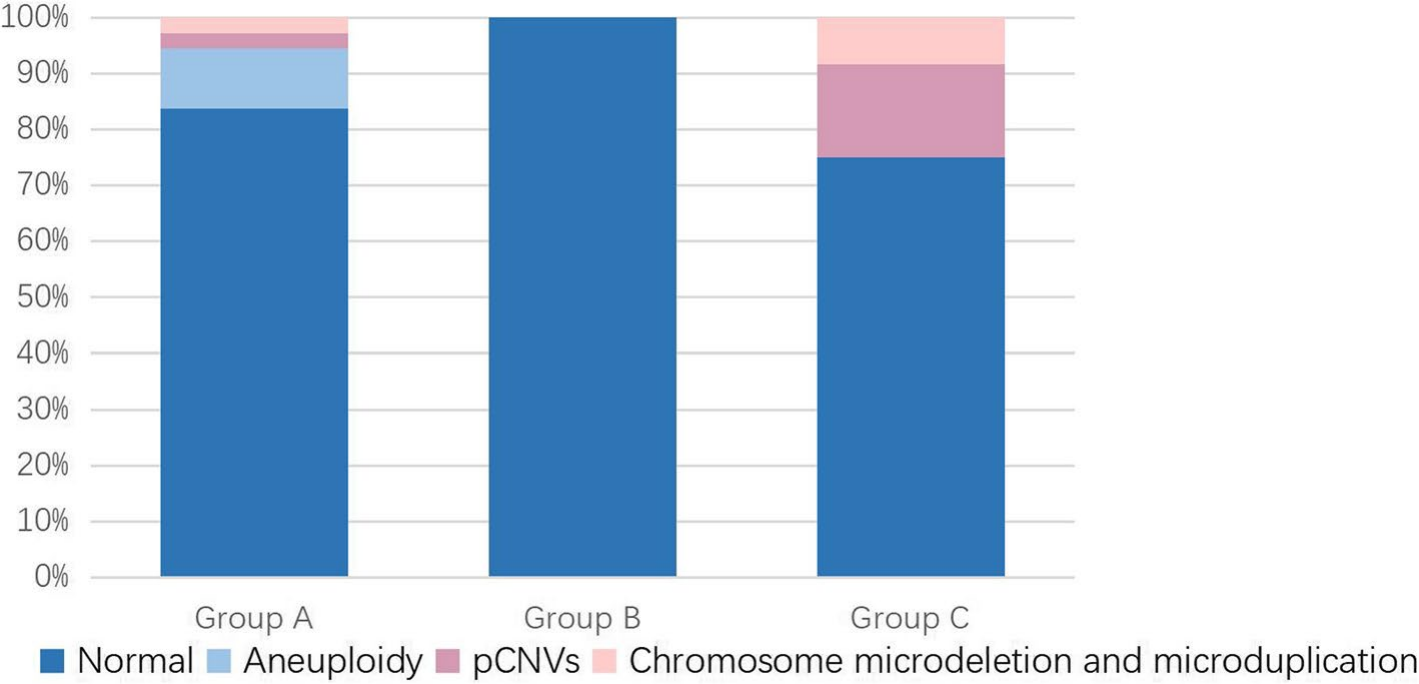
Number of chromosome results in three groups

In Group A, the rate of pathogenic findings was 16.2% (6/37), including four cases of trisomy 21, one case with pathogenic CNVs and one case with both chromosome microdeletion and microduplication. In Group B, no genomic abnormalities were found. In Group C, the rate of pathogenic findings was 25.0% (3/12), including two cases with pathogenic CNVs and one case with both chromosome microdeletion and microduplication. These results are presented in Fig. 2, Tables 1 and 2.

**Table 1:** Chromosome results of fetuses in three groups

CMA result	Normal, n(%)	Aneuploidy, n(%)	pCNVs, n(%)	Other, n(%)	Total
Group					
Group A	31(83.8)	4(10.8)	1(2.7)	1(2.7)	37
Group B	15(100.0)	—	—	—	15
Group C	9(75.0)	—	2(16.7)	1(8.3)	12

*Abbreviations*: *CMA* Chromosomal microarray analysis, *pCNVs* pathogenic copy number variations

Other: Chromosome microdeletion and microduplication

**Table 2:** Pathogenic CNVs findings in fetuses with suspected gastrointestinal obstruction

Case	GW	Prenatal imaging phenotype	CMA result	Size (Mb)
1	25	Duodenal atresia, polyhydramnios	arr[hg19]17q12(34,822,465–36,243,365) × 3	1.42
2	25	Double bubble sign, polyhydramnios, FGR	arr[hg19]7q11.23(72,414,866–74,209,949) × 1	1.8
3	34	Stomach bubbles not shown, bowel dilatation, combined multiple malformations	arr[hg19] 4p16.3p15.2(1–22,365,362) × 1	22
4	23	Double bubble sign, gallbladder not shown	arr[hg19]4p16.3(68,345–3,967,060) × 3, 12p13.33p13.31(173,786–5,406,692) × 1	3.9, 5.23
5	29	Stomach bubbles not shown, combined multiple malformations	arr[hg19]4p16.3p15.1(68,345–29,837,834) × 1, 10q25.3q26.3(118,925,004–135,426,386) × 3	29.8, 16.5

*Abbreviations*: *CNVs* copy number variations, *GW* Gestation week, *CMA* Chromosomal microarray analysis, *FGR* Fetal growth restriction

### Pregnancy outcomes and follow-up

The follow-up results of 64 fetuses with prenatal ultrasound indications of gastrointestinal obstruction include 27 live births, 3 neonatal deaths, 25 termination of pregnancy (TOP), and 9 lost to follow-up. Among 27 live births, 10 cases were not found to have gastrointestinal obstruction and the postpartum imaging was normal. 17 fetuses were diagnosed with gastrointestinal obstruction and underwent surgical treatment after birth. One of them had lower gastrointestinal obstruction combined with biliary obstruction died due to liver cirrhosis. Among three neonatal deaths, one case was esophageal atresia and two cases were unknown reason. 25 fetuses were TOP, including nine cases with chromosomal anomalies/pathogenic CNVs and 16 cases with negative CMA results (5 stillbirths, 8 induction of labor and 3 POC).

**Fig. 3:**
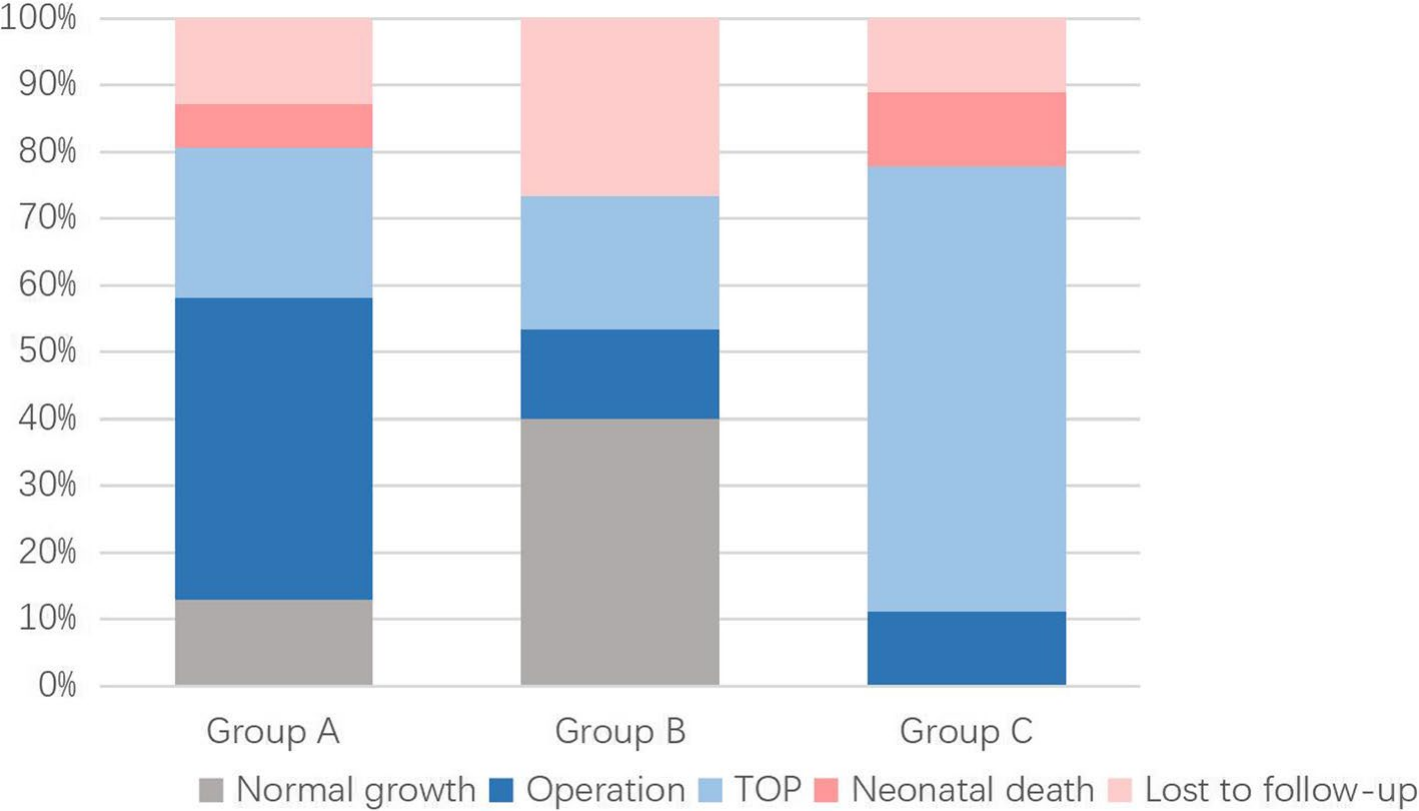
Analysis of pregnancy outcomes among three groups with normal chromosomes

The statistics of the CMA results and fetal outcomes are summarized in Fig. 3.

## Discussion

Joint Society of Obstetricians and Gynaecologists of Canada (SOGC)-Canadian College of Medical Geneticists (CCMG) recommendations suggested that CMA should be applied to fetuses with structural abnormalities [12]. CMA is recommended as a first-tier technology for prenatal diagnosis of congenital gastrointestinal obstruction. In our group, 6.3%(4/64) fetuses were diagnosed as trisomy 21. This was slightly lower than previous studies [12, 13]. This may due to application of noninvasive prenatal testing (NIPT) and improvement in ultrasonography. Additionally, submicroscopic CNVs were detected in 7.8% cases. The incidence is similar to Zhang WW’s findings [13]. The CNV findings in our study included 17q12 duplication syndrome, Williams syndrome (WS), 4p16 deletion, both 4p16 deletion and 10q duplication, both 4p16.3 duplication and 12p13.33–13.31 deletion.

Case 1, a fetus with duodenal atresia and polyhydramnios, had a 1.42 Mb duplication in chromosome 17q12. Duplication of this segment can lead to 17q12 duplication syndrome. The 17q12 duplication syndrome has mainly been proposed to include autism, behavioral abnormalities, structural brain abnormalities, learning disability, epilepsy, renal disease, atresia, and endocrine abnormalities [14–16]. Among the effected genes is *HNF1B*. Overexpression of *HNF1B* is associated with annular pancreas(AP) [17]. Although the overexpression of *HNF1B* has not been demonstrated to be directly associated with duodenal atresia, AP results in the pancreatic tissue completely or incompletely surrounding the duodenum leading to duodenal obstruction. Maria Rasmussen et al. [18] reported a patient with 17q12 duplication syndrome who was suspected “duodenal atresia” prenatally, but it was not mentioned whether the patient has been diagnosis with AP. It was a pity that autopsy was refused by the parents in our study. More researches are needed to clarify whether duodenal atresia is constitutional phenotype of 17q12 duplication or only an incidental finding.

Case 2 was a fetus with “double bubble” sign, polyhydramnios and fetal growth restriction (FGR). CMA revealed a 1.8 Mb 7q11.23 deletion, also known as Williams Syndrome (WS). This is a multisystem disorder, including but not limited to cardiovascular disease, a distinctive craniofacial appearance, and a specific cognitive and behavioral profile. Gastrointestinal obstruction was not a commonly finding of this syndrome. Previous studies [19, 20] reported a fetus of duodenal atresia who had 7q11.23 deletion. Haploinsufficiency of *ELN* gene located in this region [21] is responsible for the vascular and connective tissue features of WS [22]. Elastin haploinsufficiency could also cause hyperplasia of sub-endothelial migration and vascular smooth muscle cell, leading to encroachment on the vascular lumen and arterial stenosis [23]. Pathogenic evidence is needed to be provided to prove the causative effect of elastin deficiency and duodenal atresia.

Case 3 and case 5 were found to be absent stomach bubble, FGR and absence of nasal bone by ultrasound. 4p16 deletion was detected in both fetus by CMA. Case 5 carried a segmental duplication of 10q25. Deletions of 4p16.3 region cause Wolf-Hirschhorn syndrome (WHS). “Greek warrior helmet” face, congenital heart disease, developmental delay, hypotonia, intellectual disability and seizures are often observed in patients with WHS [24, 25]. The absence stomach bubble suggested by ultrasonography may be associated with hypotonia in patients with WHS [26, 27]. This is the first time to report absence of stomach bubble as a prenatal phenotype of WHS, in two unrelated fetuses.

Case 4 was a fetus with a 3.9 Mb 4p16.3 duplication and a 5.23 Mb 12p13.33–13.31 deletion presenting with “double bubble” sign and gallbladder not shown. So far, information about duplications of the 4p16.3 region is limited. No literature has confirmed the association between gastrointestinal obstruction and 4p16.3 deletion. The reported clinical features in patients with 12p13.3 deletions varied considerably, presumably as a result of variation in deletion size. Recurrent clinical findings in these patients include intrauterine growth retardation, schizophrenic features, muscular hypotonia, microcephaly and other congenital abnormalities [28, 29]. The deletion of 12p13.33–13.31 encompasses the *CACNA1C* and *ERC1* gene. Patients with genetic variations in the *CACNA1C* gene have been shown to have increased risk for psychiatric disorders [30]. A recent genotype–phenotype characterization proposed *ERC1*/*ELSKS* as a good candidate gene for childhood apraxia of speech (CAS). Isabela et al. [31] suggested that *ERC1* is the best candidate for the neurodevelopmental delay and autism spectrum disorders. However, neither fragment was found to be associated with gastrointestinal obstruction.

By dividing our subjects in to three subgroups, we found that the prevalence of chromosomal anomalies in non-isolated upper gastrointestinal obstruction (Group C) was about twice as high as that in isolated congenital gastrointestinal obstruction (Group A and Group B). This finding is also in accordance with Wu XQ and Meng XY’ data [32, 33]. These results proved that a detailed evaluation of the fetus and a fetal echocardiography is essential after the suscition of a GIT obstruction [34]. On the other hand, we didn’t find any genetic abnormalities in Group B. This is also observed in Orgul G’s corhort [35]. As the lower gastrointestinal obstruction is usually manifested very late in pregnancy, this information would be a great comfort for those women with such fetuses.

Postnatal outcomes of 27 live born babies in our study were generally good. Only one case of intestinal atresia combined with biliary atresia died due to liver cirrhosis. The remaining 16 babies underwent surgery at different times according to their clinical conditions. They were all alive during the follow-up period. The survival rate after surgery was 94.1%. A British study [36] showed that congenital duodenal obstruction surgery has a high success rate and a low reoperation rate. Short-term outcomes are generally good. The overall survival rate was reasonably good at 88% [37]. Other findings suggest that the long-term survival rate after surgery is more than 80% [38, 39]. Despite the progress in postnatal surgery, we should keep in mind that genetic abnormalities must be ruled out.

There are several limitations in our study. It was a retrospective study and the amount of data was small compared with other studies. Fetuses with pathogenic genetic findings were terminated without autopsy. This prevented us from further investigating the genetic-phenotype relationships. In the future, we would collect more cases and multi-center cooperation will be considered.

## Conclusion

It is crucial to understand whether the gastrointestinal tract abnormality is isolated or associated to other findings. The risk of chromosomal abnormalities in fetuses with isolated lower gastrointestinal obstruction is lower than upper gastrointestinal obstruction. While genetic abnormalities excluded, a promising prognosis is expected for fetuses with congenital gastrointestinal obstruction.

## Data Availability

All data generated or analyzed during this study are included in this published article.

## Abbreviations

CMA: Chromosome microarray analysis
GIT: Gastrointestinal tract
POC: Products of conception
ACMG: American College of Medical Genetics and Genomics
CNVs: Copy number variations
VOUS: Variants of unknown clinical significance
PCR: Polymerase chain reaction
TOP: Termination of pregnancy
SOGC: Society of Obstetricians and Gynaecologists of Canada
CCMG: Canadian College of Medical Geneticists
NIPT: Noninvasive prenatal testing
WS: Williams syndrome
AP: Annular pancreas
FGR: Fetal growth restriction
WHS: Wolf-Hirschhorn syndrome
CAS: Childhood apraxia of speech
